# Tumor Microenvironment and Its Role in Cancer Progression: An Integrative Review

**DOI:** 10.7759/cureus.92707

**Published:** 2025-09-19

**Authors:** Khalaf Mohamed Almazrouei, Vartika Mishra, Hetal Pandya, Kumar Sambhav, Sainath Narayan Bhavsar

**Affiliations:** 1 Department of College Health Sciences, Abu Dhabi University, Abu Dhabi, ARE; 2 Department of Pathology, Ruxmaniben Deepchand Gardi Medical College, Madhya Pradesh Medical Science University, Ujjain, IND; 3 Department of General Medicine, Dr. N. D. Desai Faculty of Medical Science and Research Centre, Dharamsinh Desai University, Nadiad, IND; 4 Department of Anatomy, All India Institute of Medical Sciences, Bilaspur, Bilaspur, IND; 5 Department of Engineering Sciences and Humanities, Thakur College of Engineering and Technology, Mumbai, IND

**Keywords:** cancer progression, immune evasion, precision oncology, stromal remodeling, tumor microenvironment

## Abstract

The tumor microenvironment (TME) plays a crucial role in cancer progression, metastasis, immune evasion, and treatment resistance. However, the current literature often studies its components separately. This review offers an integrated view of the dynamic interactions among fibroblasts, immune and vascular cells, the extracellular matrix, cytokines, exosomes, and microbiota within the TME. It discusses classical mechanisms such as epithelial-mesenchymal transition, stromal remodeling, and metabolic rewiring alongside emerging paradigms like microbiome-driven immunomodulation and exosome-mediated therapy resistance. Spatial heterogeneity and the temporal evolution of the tumor niche are examined using recent advances in single-cell and spatial transcriptomics, 3D bioprinting, and patient-derived organoid models. Key findings emphasize the microbiome's influence on immune responses and the role of exosomes in transferring resistance traits and regulating intercellular signaling. By connecting molecular insights with clinical perspectives, the review explores translational strategies targeting the TME, including checkpoint inhibitors, stromal modulators, anti-angiogenic agents, and engineered CAR-T therapies. This comprehensive view highlights the importance of considering cancer as a complex, evolving ecosystem rather than just a cell-autonomous disease and provides a foundational framework for precision oncology approaches aimed at disrupting harmful TME interactions to improve therapeutic effectiveness and patient outcomes.

## Introduction and background

Cancer is one of the most significant public health issues in the 21st century. According to the most recent estimates from the Global Cancer Observatory (GLOBOCAN), it resulted in almost 10 million fatalities and roughly 20 million new cases worldwide in 2024 [1]. Even though there has been an improvement in survival in certain cancers through screening and treatment, the problem of the illness worldwide, particularly in low- and middle-income nations, is still rising mainly because of late-stage disease presentation and resistance to treatment [2]. Conventionally, cancer was considered tumor-centric, whereby malignant transformation was mainly caused by mutations of oncogenes and tumor suppressor genes [3].

This model resulted in the creation of targeted therapies that target tumor cell-intrinsic drivers. Nevertheless, the growing body of clinical and experimental evidence demonstrates that these strategies are not always effective, particularly in later-stage cancers, because of the complicated interactions between cancer cells and the surrounding stroma [4].

Since 1889, the conceptual knowledge of the tumor microenvironment (TME) has been developed; Stephen Paget developed his influential theory of the soil and seed [5] in which he proposed that metastatic success is not solely dependent on the tumor cell properties (the seed) but also on the environment of the host (the soil). Fidler was able to prove this concept experimentally in the 1980s [6], showing that metastatic colonization was organ-specific and microenvironment-dependent. Hanahan and Weinberg's characteristics of cancer framework, which identified stromal support and immune evasion as enabling traits of malignancy, further established the significance of the TME [7]. The extracellular matrix (ECM), cytokines, chemokines, growth factors, and extracellular vesicles are examples of non-cellular components of the TME. Cellular components include cancer-associated fibroblasts (CAFs), endothelial cells, pericytes, adipocytes, tumor-associated macrophages (TAMs), myeloid-derived suppressor cells (MDSCs), and regulatory T cells (Tregs). To date, the TME has been identified as a highly dynamic and diverse ecosystem [8]. These elements interact with each other through a two-way communication using cytokine gradient, integrin-based adhesion, hypoxia-inducible factor (HIF), and exosome-based transfer of bioactive molecules [9].

Instead of being passive facilitators of tumors, the TME actively determines cancer behavior. As an example, stiffness and composition of the ECM control mechanotransduction pathways, which affect cell proliferation, migration, and stemness. Hypoxic tumor areas enhance metabolic reprogramming, with a preference for glycolysis and immune suppression. TAMs are often polarised to an M2-like phenotype, which promotes angiogenesis, matrix remodelling, and immune evasion in tumours [10].

**Figure 1:**
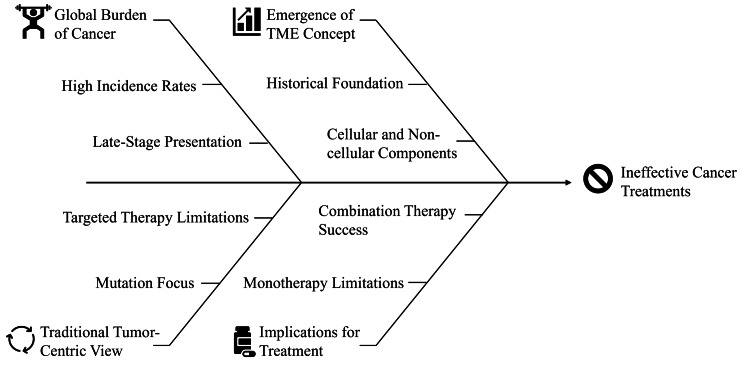
Factors contributing to ineffective cancer treatments Image credit: Khalaf Mohamed Almazrouei

This emerging concept of the TME is the reason why mono-therapies against tumor cells alone are not likely to lead to lasting responses. A classic case is pancreatic ductal adenocarcinoma (PDAC) in which drugs that inhibit immune checkpoints, including anti-CTLA-4 and anti-PD-1, have exhibited limited activity because of the dense stromal barrier and immunosuppressive microenvironment [11]. Anti-angiogenic drugs, which are directed against VEGF signaling, have initially shown promising results but have only shown temporary effects unless used in conjunction with drugs that normalize the vasculature or change immune responses [12]. On the other hand, effective combination strategies like VEGF inhibitors combined with immune checkpoint blockade have demonstrated better survival in renal cell carcinoma and hepatocellular carcinoma, which highlights the promise of TME-directed co-therapies [13]. Figure 1 illustrates the multifactorial causes of ineffective cancer treatments, including the global burden of cancer, traditional tumor-centric views, and the emergence of the TME concept.

Objectives of the review

The TME is one of the most important factors that determine tumor biology and the impact of treatment; hence, the paper offers a detailed account of the main aspects and phenomena of the TME. It examines the functions of major cellular actors, which encompass cancer-related fibroblasts (CAFs), immune infiltrates, and vascular cells and structural and biochemical components such as remodeling of ECM and metabolic reprogramming, all of which reciprocally interact with cancer cells and define tumor advancement, metastatic spread, and resistance to treatment. Meanwhile, emerging TME-focused therapeutic approaches and technologies such as single-cell transcriptomics, spatial omics, and patient-derived organoid models are transforming the ability to decipher and disrupt the cancer ecosystem. Finally, this review has also attempted to fill the gap between basic research and clinical translation by explaining how the future knowledge of the TME would lead to better prognostication, better stratification of treatment, and implementation of more personalized, sustainable, and effective cancer treatments.

## Review

TME composition

TME is a heterogeneous and dynamic ecosystem that plays an important role in tumor behavior, such as growth, immune escape, angiogenesis, and resistance to therapy [11]. It is a complex combination of both cellular and non-cellular components that have constant interaction with malignant cells. Fibroblasts, immune cells, endothelial cells, pericytes, and adipocytes are some of the cellular components that play a major role within the stroma of the tumor as they bestow individually to the biochemical and biomechanical characteristics of the tumor stroma [12]. The fibroblasts, particularly those that have been converted into CAFs, are the key producers of ECM proteins and paracrine factors [13]. Endothelial cells and pericytes are essential to the development and stabilization of tumor vasculature and, therefore, affect perfusion and metastatic capability. Adipocytes, especially those found in the adipose-rich organs like the breast and ovary, provide lipids and signaling molecules that favor tumor progression [14].

The TME's non-cellular constituents, particularly the ECM, are also important in determining tumor behavior. The ECM not only offers structural support, but it also modulates cellular behavior by controlling adhesion, polarity, and receptor-mediated signaling. The ECM is made up of laminins, collagens, fibronectin, and hyaluronan and is continuously remodelled in the development of tumors, with matrix metalloproteinases (MMPs) and LOX as prominent enzymes [4]. These alterations increase the rigidity of the matrix, alter the tissue structure, and provide physical barriers to drug penetration, which all lead to therapeutic resistance [15]. The ECM is also a storehouse of soluble factors released in the process of degradation, which form the gradients that guide the migration of the cell and the development of angiogenesis.

A cytokine, chemokine, and growth factor network (such as TGF-β, VEGF, interleukins, and CXCL12) creates a core signaling axis in the TME and regulates the conversation between neoplastic and stromal cells [16]. Such signaling maintains inflammation and inhibits productive immune surveillance. As an example, CAF-secreted CXCL12 paracrine loop on cancer cell CXCR4 has been demonstrated to induce immune cell exclusion and the metastatic spread [17]. Such signalling dynamics are additionally confounded by the spatial heterogeneity in the TME. Areas of hypoxia tend to overlap with thick ECM and immunosuppressive niches, which favor the glycolytic adaptation, neoangiogenesis, and the exclusion of T cells. Conversely, perivascular niches could contain infiltrating immune cells and stem-like cancer cells to form microdomains that possess different phenotypes and drug sensitivities [18].

Such cellular heterogeneity in terms of cellular composition, vascularization, and metabolic gradients causes high functional diversities in tumors. The interstitial fluid pressure, oxygen tension, and the availability of nutrients are not only different across the tumors but also within the same lesion. Such gradients affect the process of clonal evolution and response to treatment, which is why the need to consider the development is impacted by the intricacy of TME's therapeutic strategies [19].

Cancer-associated fibroblasts (CAFs)

Among the most prevalent and diverse cell types in the TME are cancer-associated fibroblasts (CAFs). Pericytes, resident fibroblasts, mesenchymal stem cells, and epithelial or endothelial cells that go through transdifferentiation by the epithelium-to-mesenchymal transition (EMT) or endothelium-to-mesenchymal transition (EndMT) are some of their diverse sources [20]. Activation signals (e.g., TGF-β, PDGF, and IL-1) generated by tumor cells promote the precursor populations to a myofibroblastic phenotype. It is frequently stated that CAFs express platelet-derived growth factor receptor beta (PDGFRB), fibroblast activation protein (FAP), and α-smooth muscle actin (alpha-SMA) [21]. These CAFs are activated and can take various effector functions that remodel the physical and immunological structure of the tumor.

A principal function of CAFs is remodeling the ECM. They produce and accumulate structural proteins, including collagen I and fibronectin, as well as producing crosslinking enzymes like LOX and proteases like MMP-2 and MMP-9 [22]. This kind of remodelling makes the ECM stiffer and creates parallel collagen fiber that acts as a route of migration for invasive cancer cells. This mechano-strengthened ECM also promotes integrin signalling that promotes epithelial plasticity and triggers EMT of neighboring tumor cells [23]. CAF activity extends well beyond ECM structuring. They release multiple growth factors, including VEGF, HGF, FGF2, and IGFs, that sustain the development and angiogenesis of tumor cells, and also sustain tumor cells in a stressful environment. Their secretome is also very influential on how the immune dynamics play out in the TME. CAFs produce CXCL12 to create chemokine gradients that physically exclude CD8+ + T cells from tumor nests [10]. They also release TGF-β, IL-6, and prostaglandin E2, which are agents that recruit immunosuppressive myeloid cells and stimulate their functional polarization, but they prevent T cells and NK cells from maturing and killing [24].

**Figure 2:**
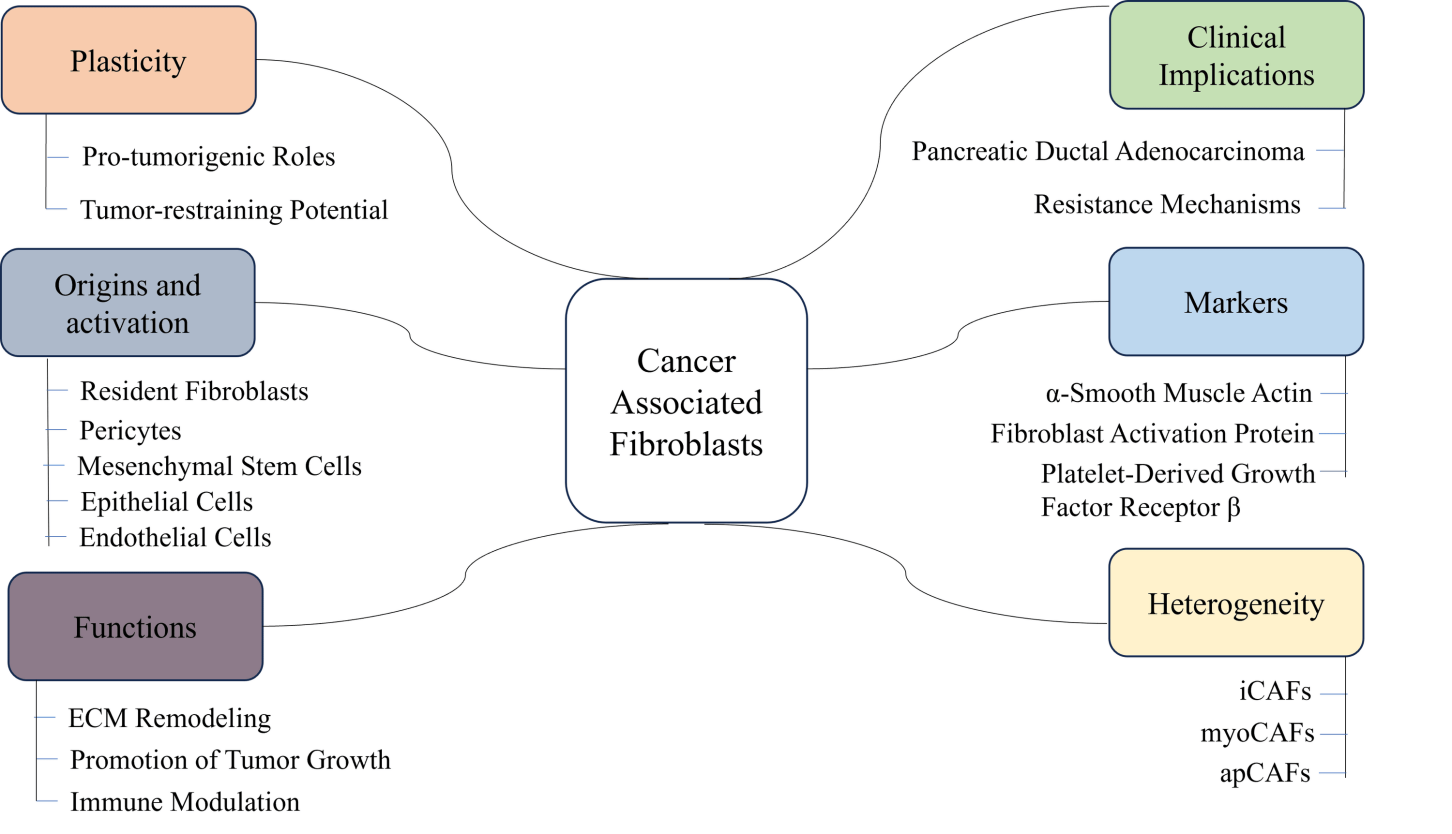
Fibroblast roles in the tumor microenvironment Image credit: Khalaf Mohamed Almazrouei

Recent single-cell transcriptomic studies have demonstrated high heterogeneity of the CAF compartment. Separate subsets such as inflammatory CAFs (iCAFs), myofibroblastic CAFs (myoCAFs), and antigen-presenting CAFs (apCAFs) have different secretory patterns and functions in ECM production and immune modulation [25]. iCAFs mainly produce cytokines and modulate the chemotaxis of immune cells, whereas myoCAFs are more oriented toward ECM and contractile. apCAFs, which are less studied yet, seem to be able to present antigens. It is notable that in recent evidence, there is a possibility that CAFs also possess tumor-restraining abilities in certain circumstances, which highlights the plasticity of their functionality [9]. Nonetheless, their overall influence in most malignancies is pro-tumorigenic. As an example, in pancreatic ductal adenocarcinoma, CAFs generate dense desmoplastic stroma containing a mechanical barrier and a signaling hub, which limits the effectiveness of chemotherapy. On the same note, CAF-induced STAT3 and PI3K/AKT activation has been found to induce targeted therapy and immunotherapy resistance in breast and lung cancers [26]. Figure 2 depicts the key attributes of CAFs, including their diverse origins, molecular markers, functional roles in tumor progression, phenotypic heterogeneity, clinical implications, and functional plasticity within the TME.

Immune landscape within the TME

The TME is an immune contexture that consists of the adaptive and the innate immune cells, the phenotype and functions of which are regulated by tumor signals and interactions with stroma. Specifically, the epicentre of anti-tumor immunity is tumor-infiltrating lymphocyte (TIL), which kills cancer cells during the mechanism of MHC class I restricted antigen presentation [26]. Nonetheless, in case these T cells are stimulated persistently by antigens and in the case when they are exposed to immunosuppressive cytokines such as this, this tends to create a situation where these T cells end up being functionally exhausted (i.e., upregulating their receptors that inhibit the effect, such as PD-1, TIM-3, and LAG-3) [12]. CD 4+ T helper cells, and more specifically the Th1 cells, contribute to anti-tumor response through secretion of interferon-gamma and augmented antigen presentation. Conversely, the Tregs that are characterized by FOXP3 expression are immunosuppressive and act through IL-10, TGF-β, and CTLA-4 pathways. There is also a potential indicator of low response to immunotherapy and poor prognosis, which is a high Treg/CD8+ T ratio [17]. Tumor-associated macrophages (TAMs) are a highly plastic cellular group that takes a phenotype in response to signals received by TME. TGF-β, IL-10, and IL-4 also cause TAMs to polarize toward an M2-like phenotype that allows immune suppression, angiogenesis, and matrix remodeling [15]. These macrophages secrete VEGF, IL-10, and MMPs but also checkpoint molecules such as PD-L1 and VISTA with direct inhibitory effects on T cell effector functions. Tumoricidal and pro-inflammatory M1-polarized TAMs are normally restricted to early-stage tumors and expelled in the disease by M2 TAMs as the disease advances [20]. The GM-CSF and IL-6 attract myeloid-derived suppressor cells (MDSCs), which contain monocytic and granulocytic subsets, to the TME. Once inside the tumor, they suppress the functions of T cells and NK cells by synthesizing nitric oxide, reactive oxygen species, and arginase-1. MDSCs also hinder DCs' maturation and antigen presentation, further lowering adaptive immunity. They are linked to checkpoint inhibitor resistance and poor clinical outcomes in a number of cancers [27]. The tumor milieu is also influenced by immune cells. The natural killer (NK) cells are very powerful in theory and may be inactivated in the tumor by TGF-β, indoleamine 2,3-dioxygenase (IDO), and inhibition of activating ligands [5]. Immune escape results from immature or functionally tolerogenic dendritic cells in the TME, which are crucial for priming T cell responses [16]. Immune and non-immune cell interaction (e.g., CAFs and endothelial cells) is also a significant determinant of immune cell localization and phenotype. As an example, CXCL12 gradients made by CAF have been revealed to keep or kill effector T cells out of tumor nests, as well as endothelial cells expressing FasL. In general, the immune microenvironment is a dynamic effector-suppressive balance [28]. The immune editing process (elimination, equilibrium, and escape) results in the selection of tumor variants that are resistant to immune recognition, MHC downregulation, or antigen loss. The therapeutic success is now strongly based on our capability to balance this, transforming immunologically cold tumors into hot and re-establishing local immune suppression by rational combinatorial approaches [17].

Hypoxia and metabolic reprogramming

Solid tumor also has a characteristic of hypoxia as a result of the cells growing at a rate faster than the supply of vasculature. Such low oxygen concentration stabilizes the transcriptional factor HIF-1 due to the decreased activity of the prolyl hydroxylases in oxygen and the fact that this transcription factor cannot be degraded [26]. The stabilized HIF-1 that is translocated to the nucleus triggers the expression of the gene that is responsible for glucose metabolism, angiogenesis, immune modulation, and survival [27]. One of the most relevant roles of HIF-1alpha includes aerobic glycolysis or the Warburg effect [28]. Even when they are under oxygen, tumor cells will utilise glycolysis in preference to oxidative phosphorylation to produce ATPs and biomass rapidly. This leads to the buildup of lactate, which contributes to the extracellular acidification and the local tissue remodeling [29].

**Figure 3:**
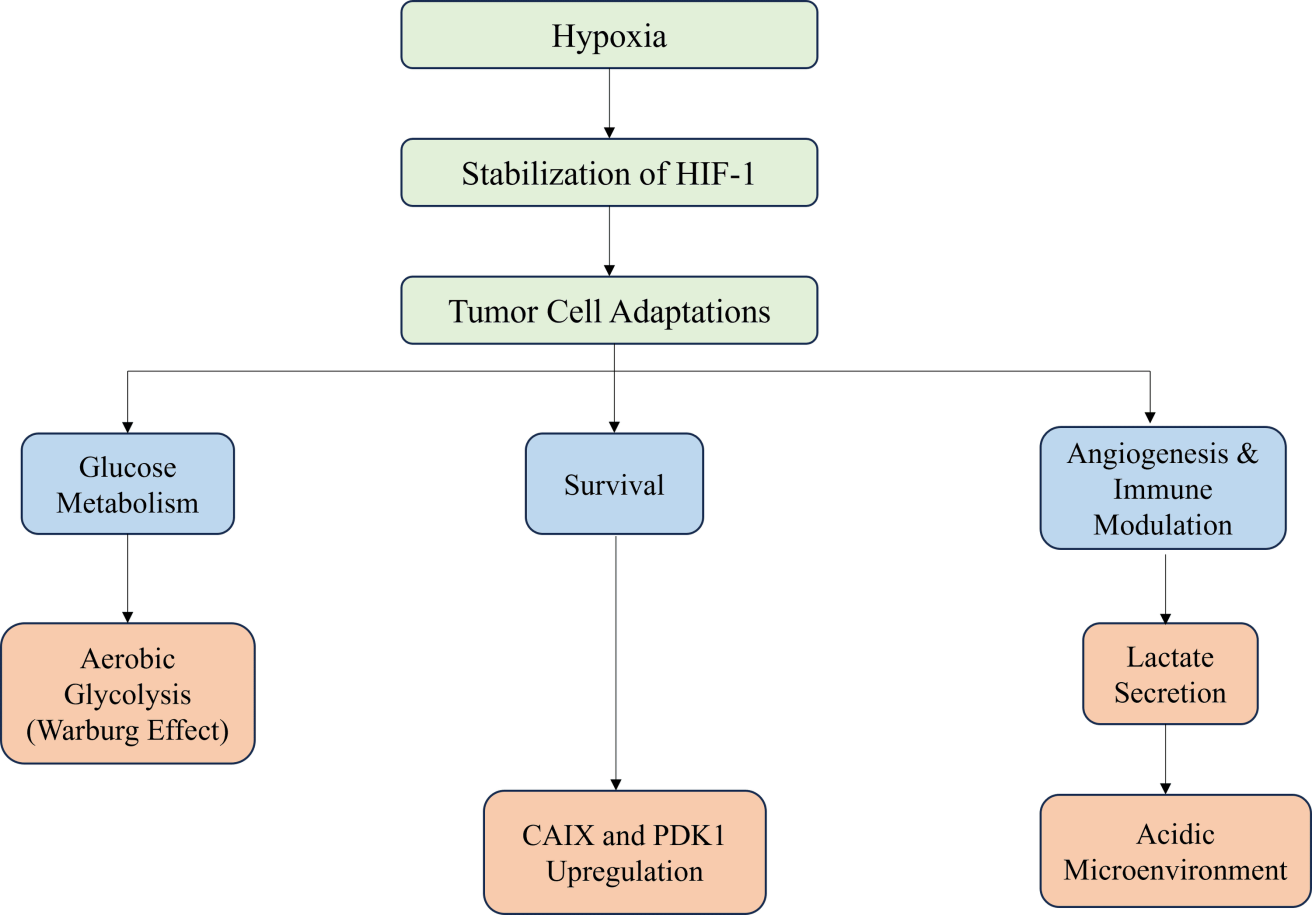
Hypoxia-induced adaptations in tumor cells Image credit: Khalaf Mohamed Almazrouei

An acidic environment also enhances the activity of the MMPs and suppresses the activity of the cytotoxic T cells and NK cells, which prevents immune evasion [30]. In isolation, lactate is a signaling molecule that induces M2 polarization of macrophages and induces VEGF expression by endothelial cells, thereby promoting angiogenesis and immunosuppression [31]. Additional enzymes that are induced by HIF-1α are carbonic anhydrase IX (CAIX) and pyruvate dehydrogenase kinase 1 (PDK1), which promote intracellular pH homeostasis and inhibit mitochondrial respiration [32]. Additional adaptation of tumor cells to prolonged hypoxia is an increased glutamine uptake and glutaminolysis activation to maintain the TCA cycle and redox homeostasis [28]. These metabolic adaptations not only enhance the survival of the tumor but also predispose it to therapeutic resistance and heterogeneity [14]. Several HIF-1 alpha inhibitors and metabolic modulators are at preclinical and early clinical stages as potential treatment strategies [5]. Figure 3 shows the adaptive mechanisms in tumor cells caused by hypoxia that involve stabilization of HIF-1, which, in turn, affects aerobic glycolysis, lactate production, angiogenesis, immune modulation, CAIX upregulation, and PDK1 upregulation, resulting in tumor cell survival and the TME becoming more acidic.

Tumor angiogenesis and vasculature remodeling

Artificial angiogenesis is essential to tumor growth to a level above minimal and is mostly dependent on VEGF expression brought about by hypoxia [3]. VEGF combines with VEGFR2 on endothelial cells and induces proliferation and the creation of new capillaries [1]. Tumor blood vessels that grow under such stimulation are structurally abnormal; vessels are inadequately covered by pericytes, convoluted, and leaky [33]. The abnormalities lead to an uneven perfusion, chronic hypoxia, and high interstitial pressure, which all cause immune cell exclusion and drug delivery inefficiency [18]. Although anti-angiogenic VEGF/VEGFR agents have demonstrated clinical value in a few cancers, they are usually short-lived since they induce a compensatory upregulation of other pro-angiogenic mediators like FGF2 and IL-8 [34]. Other mechanisms that are independent of endothelial cell phenotype proliferation that may be used by tumors to escape VEGF inhibition include vessel co-option and vascular mimicry [31].

The hypoxia may be aggravated by aggressive pruning of tumor vessels, which may enhance invasion through EMT [28]. In reaction, the term vascular normalization has appeared, which is intended to develop functional vessel architecture with better perfusion [35]. Anti-angiogenic therapy, when dosed correctly, temporarily normalizes vasculature, increases drug delivery, and improves immune cell infiltration [32]. This approach supports the basis of the combination of VEGF inhibitors with checkpoint blockade, which was reflected in clinical benefit with atezolizumab and bevacizumab combination in hepatocellular carcinoma [36]. Angiogenesis, thus, is not only a growth facilitator, but it is also a gateway or an adjustable barrier to other treatments. It is important to balance between vessel suppression and restoration to achieve successful modulation.

ECM remodeling

Tumor ECM is a thick and restructured network of collagens, fibronectin, laminins, and hyaluronan [17]. It not only gives structural integrity but also gives biochemical cues that regulate cancer progression [26]. CAFs are primary mediators of ECM remodeling. They also produce fibrillar collagens, tenascin-C, and enzymes that provide crosslinks, i.e., lysyl oxidase (LOX), that enhance the stiffness of the matrix [20]. The physical stiffening reinforces integrin-mediated signaling in tumor cells, particularly via the downstream PI3K/AKT and YAP/TAZ pathways and focal adhesion kinase (FAK) [30]. ECM stiffness induces EMT, enhances the motility of cancer cells, and leads to apoptosis/chemotherapy resistance [31]. Furthermore, a rigid matrix causes compressed vasculature that is the cause of chronic hypoxia, which strengthens angiogenesis and metabolic stress [18]. Both tumor cells and stromal fibroblasts secrete MMPs (MMP-2, MMP-9), which degrade the components of the ECM and release the latent growth factors, including VEGF and TGF-β [29].

These molecules also induce angiogenesis, modulation of immunity, and activation of fibroblasts. Integrin-mediated mechanotransduction forms a positive feedback mechanism-tumor cells detect ECM rigidity and increase their expression of MMPs and LOX, continuing the process of ECM remodeling [37]. The spatial disparity in the makeup of the matrix results in the development of the invasive tumor fronts that have a specific biomechanical character. Treatment of ECM is therapeutically difficult because ECM is physiologically essential to normal tissues. Nonetheless, some of the investigational approaches are LOX inhibitors, integrin blockers, and hyaluronidase-based approaches that decrease the stiffness of the matrix and enhance the delivery of treatment [25]. PXS-5382A is one such agent in early-phase testing in desmoplastic tumors [34]. Tumors alter their structural microenvironment by reprogramming the ECM, as well as creating signaling environments that become resistant to immune attack and restrict therapy. ECM stiffness and remodeling are, therefore, another avenue of complementing the efficacy of immunotherapy and drugs.

Exosomes and intercellular communication

Exosomes are nano-sized (30-150 nm) vesicles released by cancer cells, fibroblasts, endothelial cells, and immune cells in the TME [38]. Those vesicles are active agents of intercellular communication, which deliver bioactive fragments that can mold the conduct of recipient cells [14]. The contents of exosomes, exosomal cargo, are selectively packaged microRNAs (miRNAs), messenger RNAs, proteins, lipids, and fragmented DNA that capture the state of metabolism and signaling of the donor cell [39]. The profiles of exosomes are altered under stress conditions like hypoxia to favor malignancy and immunosuppression [26]. As an example, the tumor-secreted exosomes that are loaded with miR-21 or miR-210 stimulate angiogenesis, as well as suppress immune activation in target cells [40].

Exosomes aid metastasis by creating pre-metastatic niches in remote organs. The deposition of fibronectin, immune suppression, and raised vascular permeability by these vesicles reprogram stromal cells to be receptive microenvironments of future metastatic sites by reprogramming the stromal cells [41]. Tumor-associated fibroblasts and macrophages' exosomes may also be used to stimulate EMT in cancer cells, increasing motility and invasiveness [18]. Exosomes containing ligands like PD-L1 help patients evade the immune system by binding to PD-1 on CD8+ T cells and stopping their cytotoxicity [42]. In addition, IDO and other enzymes found in other exosomes locally consume tryptophan and prevent T cell growth [30]. Others release TGF-β, which tilts the immune system in favor of regulatory T cell development and NK cell cytotoxicity avoidance [43].

Exosomes also mediate resistance to therapy. They can pass drug-efflux pumps and resistance-related miRNAs to the formerly sensitive cells, fostering acquired resistance [25]. At the same time, exosomes can capture and trap chemotherapeutic agents or therapeutic antibodies in the circulation and decrease the efficacy of drugs [44]. Due to their systemic access and stability at the molecular level, exosomes are also examined more as a biomarker in a liquid biopsy and as a delivery vehicle of therapeutic cargo. Nevertheless, clinical translation is still difficult because cell-specific targeting and off-tumor effects have to be avoided [13]. Table 1 categorizes exosomes based on their cell of origin within the TME, detailing their molecular cargo, target interactions, and downstream effects.

**Table 1:** Functional roles and clinical implications of tumor-derived exosomes in cancer progression Table credit: Khalaf Mohamed Almazrouei

Source cell type	Exosomal cargo	Target cell	Mechanism of action	Functional outcome	Clinical relevance	Limitations	References
Tumor cells	miR-21, miR-210, PD-L1, TGF-β	Endothelial cells, immune cells, and stromal cells	Angiogenesis stimulation, immune checkpoint inhibition	Immune suppression, enhanced vascularization	Liquid biopsy biomarkers, immune therapy prediction	Off-tumor immune suppression	[40]
Cancer-associated fibroblasts (CAFs)	Tenascin-C, matrix-modulating enzymes, exosomal miRNAs	Tumor cells, extracellular matrix	ECM stiffening, EMT promotion	Invasiveness, resistance to therapy	Monitoring of tumor aggression	Exosome content overlaps with healthy fibroblasts	[18]
Tumor-associated macrophages	IDO, PD-L1, TGF-β	CD8+ T cells, NK cells	T cell suppression, regulatory T cell differentiation	Immune evasion	Target validation for immunomodulatory therapy	Systemic immunosuppression via exosomes	[30]
Hypoxic tumor cells	Hypoxia-induced miRNAs (e.g., miR-210), stress-related proteins	Stromal and endothelial cells	Modulation of metabolism and vascular responses	Pro-angiogenic and immunosuppressive shift	Marker for tumor hypoxia and aggressiveness	Dynamic changes under stress complicate analysis	[26]
All TME cells	Fragmented DNA, lipids, and heat shock proteins	Pre-metastatic niches (e.g., liver, lung)	ECM preconditioning, vascular permeability elevation	Favorable microenvironment for metastasis	Predictive marker for metastasis	Standardization in detecting organotropism	[20]
Resistant tumor cells	Drug-efflux pumps (e.g., P-gp), miRNAs (e.g., miR-155, miR-222)	Previously sensitive tumor cells	Horizontal transfer of resistance traits	Spread of drug resistance	Stratification for chemotherapy failure risk	Difficult to block intercellular exosome transfer	[25]
Engineered exosomes	siRNAs, CRISPR-Cas9, anti-oncomiRs, chemotherapy agents	Tumor cells (specific targeting attempted)	Ligand-based targeting, intracellular delivery	Precision gene modulation or drug delivery	Future therapeutic platforms	Targeting precision and biodistribution challenges	[13]
Circulating exosomes	Tumor antigens, EpCAM, HER2, integrins	Blood (for sampling), target organs	Diagnostic capture, organotropic migration	Early cancer detection, metastatic site prediction	Basis for non-invasive diagnostics	Sample handling, inter-patient variability	[44]

EMT and invasion

E-cadherin down-regulation, polarity loss, and enhanced mobility are all components of the reversible biological conversion of epithelial cells into mesenchymal cells, known as the EMT [2]. In cancer, EMT encourages local invasion, metastasis, and resistance to immunological and therapeutic pressure [45]. The TME provides the signals required to initiate and sustain EMT. Transforming growth factor-beta (TGF-β) is a potent inducer that activates SMAD signaling pathways, which suppress epithelial traits and activate genes in the skin [40]. Tumor cells and stromal elements also secrete interleukin-6 (IL-6) that supports EMT via the STAT3 signaling pathway and maintains a pro-invasive state [8].

Hypoxia also enhances EMT through stabilization of HIF-1alpha, which teams up with transcription factors such as SNAIL and TWIST and coordinates the expression of EMT-related genes [26]. Such transcriptional modifications modify the cytoskeleton structure and increase the invasiveness of tissues [46]. EMT is closely linked to stemness and plasticity. The CD44, ALDH1, and Nanog markers are expressed by EMT-induced cancer cells, which makes them part of the tumor heterogeneity and self-renewal ability [41]. These are mesenchymal-like cells that are frequently enhanced following treatment by chemotherapy or radiotherapy and are involved in tumor recurrence [22].

EMT also enables immune evasion. MHC class I is down-regulated and immune checkpoint ligands such as PD-L1 are up-regulated in mesenchymal tumor cells, limiting their destruction by cytotoxic T lymphocytes [43]. Moreover, EMT can transform the microenvironment of the area through enhanced production of cytokines, which attract immunosuppressive myeloid cells [10]. Nevertheless, EMT targeting can be a feasible therapeutic objective, even though it is reversible and context-specific. Preclinical data have demonstrated the potential of TGF-β signaling inhibitors, including galunisertib, in interrupting invasion and resistance mediated by EMT [47]. These pathways are under investigation in order to develop ways of preventing metastasis and long-term cancer control.

Tumor microbiome and its crosstalk

Tumor microbiome is a microbial community consisting of intratumoral bacteria, fungi, and viruses and has been demonstrated to be a crucial modulator of cancer biology [48]. These microorganisms that are found inside the tumor cells, the immune populations, and stromal niches engage with the signaling networks of the host and modify the tumor progression [3]. In the last 10 years, intratumoral microbes have been detected in colorectal, pancreatic, breast, and other cancers [49]. They influence immune modulation, cytokines, and the distribution of TILs [20]. As an example, *Fusobacterium nucleatum* in colorectal cancer enhances tumor development via the activation of the NF-κB and suppression of adaptive immunity [13].

Microbial metabolites and enzymes also interfere with cancer therapy. Some of the bacterial species, such as *Gammaproteobacteria*, have cytidine deaminase enzymes that break down gemcitabine and cause pancreatic cancer to be resistant to chemotherapy [26]. On the other hand, beneficial microbes improve the checkpoint inhibitor responses through the regulation of dendritic cell maturation and T cell priming [28]. Microbes present intratumorally help in metastasis through reprogramming of the stromal cells in the remote organs, stimulating the production of fibronectin and CXCL12 and vascular leakage [20]. These modifications precondition accommodating pre-metastatic niches before the arrival of circulating cancer cells [41].

Microbes also affect the differentiation of myeloid cells and polarization of macrophages to a tumor-promoting M2 phenotype in the immune context [45]. They also influence the density and activity of neutrophils and dendritic cells that determine the local immunity and tumor inflammation [30]. The clinical potential of microbiome manipulation is growing. Microbial depletion enhances CD8+ T cell infiltration in pancreatic cancer models, as well as rendering tumors sensitive to PD-1 blockade [49]. Microbial profiles in breast cancer were linked to chemotherapy- and immune checkpoint therapy-differential responses [42]. There is an attempt to apply microbial profiling as a prognostic marker and design microbiome-altering agents that re-immunogenize TME [48]. These methods are under investigation in cohorts of colorectal and melanoma patients to increase immune responsiveness and inform treatment choice. Table 2 shows how tumor-associated microbes influence cancer progression, immune modulation, metastasis, and therapy response through diverse mechanisms.

**Table 2:** Functions of the tumor microbiome in the development and treatment of cancer response Table credit: Khalaf Mohamed Almazrouei

Microbial role	Microbial group	Associated cancer type(s)	Mechanism of action	Clinical outcome	Therapeutic relevance	References
Immune suppression and NF-κB activation	Fusobacterium nucleatum	Colorectal cancer	Activates NF-κB, suppresses adaptive immunity	Promotes tumor progression and poor immune infiltration	Potential target for microbiome-modulating therapy	[13]
Chemotherapy resistance	Gammaproteobacteria	Pancreatic cancer	Cytidine deaminase inactivates gemcitabine	Induces chemoresistance	Suggests the need for pre-treatment microbial screening	[26]
Enhancement of checkpoint inhibitor response	Beneficial commensal microbes	Melanoma, other solid tumors	Stimulate dendritic cell maturation, T cell priming	Improved immunotherapy efficacy	Basis for microbial adjuvants	[28]
Immune cell modulation	Various tumor-resident microbes	Multiple cancers	Regulate cytokine levels and distribution of tumor-infiltrating lymphocytes (TILs)	Immune context shift	Influences prognosis and therapy response	[20]
Pre-metastatic niche formation	Unspecified intratumoral microbiota	Breast, colorectal, and other cancers	Stimulates fibronectin and CXCL12 production, increases vascular permeability.	Enhances metastatic potential	Target for metastasis-prevention strategies	[41]
Macrophage polarization	Diverse tumor-associated microbes	Pancreatic, colorectal, and breast	Skews macrophages toward an M2 tumor-promoting phenotype	Immunosuppressive TME	Supports reprogramming efforts (e.g., immune-modulating bacteria)	[45]
Myeloid and dendritic cell differentiation	Tumor-resident bacteria/fungi	Multiple	After differentiation and activation of DCs, neutrophils	Impacts antigen presentation and local inflammation	Affects checkpoint therapy outcomes	[30]
Tumor infiltration enhancement by depletion	Microbial depletion	Pancreatic cancer	Decreases immunosuppression, allowing CD8+ T cell infiltration	Increases sensitivity to PD-1 blockade	Microbiome depletion as a co-therapy	[29]
Microbial signature–based treatment prediction	Tumor-specific microbial profiles	Breast cancer, melanoma, and CRC	Correlates with response variability to chemotherapy and immunotherapy	Enables prognostic stratification	Microbial biomarkers in precision oncology	[42]
Prognostic tool development	Global tumor microbiome composition	Colorectal, melanoma	Profiling is used to identify favorable vs. suppressive microbial ecosystems.	Treatment tailoring and outcome prediction	Microbiome-based diagnostics and treatment customization	[18]

Therapeutic targeting of the TME

Because it encourages immune suppression, invasion, and treatment resistance, inhibiting the TME has become one of the most important cancer strategies [1]. Immune checkpoint drugs that restore T cell effector function by blocking PD-1, PD-L1, or CTLA-4 are among the most extensively studied and clinically ground-breaking treatments [12]. The checkpoint blockade has demonstrated sustained response in melanoma, non-small cell lung cancer (NSCLC), and renal cancers, yet limited efficacy in cold tumors that are characterized by dense stroma and lack of immune infiltration [27]. Combination strategies in such environments are meant to transform the immunologically silent tumors into responsive ones.

The VEGF pathway is blocked using anti-angiogenic drugs, including bevacizumab and ramucirumab, to inhibit the development of abnormal vessels, alleviate hypoxia, and deliver drugs and immune cells [18]. Nevertheless, there is a tendency toward resistance through VEGF-independent angiogenesis and immune evasion with monotherapy [29]. The normalization of vasculature, which is accomplished due to optimal dosing of anti-angiogenic agents, increases tissue perfusion and decreases hypoxia, which increases the efficacy of immunotherapies and chemotherapeutics [34]. There have been improved results with clinical studies that combine VEGF inhibitors with checkpoint blockade, atezolizumab, and bevacizumab in hepatocellular carcinoma [50]. The other important components of TME are CAFs, which promote immune exclusion and ECM remodeling, being obstacles to therapy [22]. Agents that act on FAP, TGF-β, or CXCL12 are in development to correct the behavior of CAF and boost immune infiltration [39].

While CAR T cells have demonstrated efficacy in hematological malignancies, stromal barriers, inhibitory cytokines, and nutritional restriction have hindered their ability to function in solid tumors [46]. CAR-T cells are being developed to produce two enzymes, such as heparanase or hyaluronidase, to break down ECM and inhibit TGF-β signaling in order to get around these restrictions [49]. Nonetheless, spatial heterogeneity, poor drug distribution, and compensatory resistance can be a major cause of TME-targeting strategies [25]. Concurrent immune, vasculature, and stromal architecture modulation combination therapies are on the rise in clinical trials [50]. In the future, the TME will need to be attacked multi-modally, with metabolic, immunologic, and stromal manipulations, to enhance treatment durability and realize the goals of personalized cancer control.

Limitations and future recommendations

Even though there have been considerable developments in the field of TME, a number of constraints still impede translation. The cellular heterogeneity of the human TME, biomechanical forces, and immune dynamics are not well captured in conventional in vitro systems and animal models [1]. Therefore, they tend to produce outcomes that cannot be translated into human trials because of the differences in models and systems. The TME is also spatially and temporally heterogeneous, such that, depending on the cancer type, and even within a given tumor, stromal architecture, immune cell infiltration, and metabolic gradients may vary [14]. The complexity is added to the fact that translational issues are complicated by the inability to define robust and quantifiable endpoints, i.e., stromal reprogramming or immune cell infiltration, that would reflect changes in TME in real time [26]. In addition, the molecular signaling networks and interdependent feedback loops that drive TME-tumor interactions are not fully described, and inhibit the rational, pathway-specific combination treatment.

Single-cell and spatial omics technologies provide higher-resolution tools to deconvolve the complexity of TME and allow mapping cellular heterogeneity, intercellular communication, and spatial anatomy at a high resolution [39]. Such methods have the potential to reveal uncommon yet functionally vital subpopulations, e.g., immunoregulatory dendritic cells or therapy-resistant fibroblast subsets. Simultaneously, patient-derived organoids and 3D bioprinted tumor models are developed that allow for the study of tumor-stroma interactions and test therapies in physiologically relevant conditions [51]. TME profiling- Individualized TME profiling, including immune cells, stromal markers, and metabolic signatures, could inform precision medicine approaches based on the TME. Therapeutic approaches combining immune checkpoint inhibitors, stromal, and metabolic targeting have been of special interest in overcoming mono-therapy resistance [52]. In the future, longitudinal studies following the evolution of the TME during the development of the disease and therapy will be important to predict the resistance mechanisms and develop adaptive treatment schedules with greater clinical sustainability.

## Conclusions

The TME is no longer viewed as a passive backdrop but as a dynamic, co-evolving ecosystem that decisively governs tumor behavior. This review integrates current evidence on both cellular (e.g., CAFs, immune cells, endothelial elements) and non-cellular (e.g., ECM, hypoxia, microbiota) components of the TME, highlighting how their crosstalk orchestrates tumor progression, immune evasion, and resistance to treatment. By connecting well-established mechanisms with emerging paradigms such as the role of the tumor microbiome, exosomal communication, and metabolic symbiosis, this review offers a holistic perspective on TME-driven malignancy that transcends tumor-centric models.

Importantly, this synthesis emphasizes the translational implications of TME biology by outlining how cutting-edge technologies like single-cell and spatial omics, 3D bioprinting, and patient-derived organoids can resolve intra-tumoral heterogeneity and facilitate patient-specific therapeutic strategies. The review proposes that future success in oncology will depend not only on targeting tumor-intrinsic vulnerabilities but also on actively reprogramming the TME to support immune and therapeutic engagement. In doing so, it provides a conceptual and practical roadmap for researchers and clinicians seeking to develop more durable, personalized, and combination-based approaches to cancer therapy.
